# Impact of oxidative stress on myocardial damage visualized by cardiac resonance imaging in acute ST-elevation myocardial infarction

**DOI:** 10.1186/1532-429X-17-S1-P99

**Published:** 2015-02-03

**Authors:** Georg Fuernau, Steffen Desch, Suzanne de Waha, Gerhard Schuler, Volker Adams, Holger Thiele, Ingo Eitel

**Affiliations:** University of Leipzig - Heart Center, Leipzig, Germany; Department of Internal Medicine/Cardiology/Angiology/Intensive Care Medicine, University Hospital Schleswig-Holstein, Campus Lübeck, Lübeck, Germany; Heart Center Bad Segeberg, Bad Segeberg, Germany

## Background

In acute ST-elevation myocardial infarction (STEMI) oxidative stress is an important determinant of severe myocardial damage and reperfusion injury. However, no data are currently available about the correlation of oxidative stress with cardiac magnetic resonance (CMR) markers of myocardial injury in acute STEMI.

## Methods

We studied 198 patients with acute STEMI undergoing primary percutaneous coronary intervention (PCI) within 12 hours of symptom onset. Oxidative stress was determined by oxidated LDL-cholesterol (oxLDL), activated oxygen protein products (AOPP) and lipid hydroperoxides (perOx). Blood samples were collected before PCI, after PCI and on day 2 as well as day 3 and 4. Follow-up values were expressed as ratio to baseline values. CMR studies were performed 2-4 days after the infarction (median 3 days, interquartile range 2, 4 days) using a standard infarction protocol.

## Results

A rise in oxidative stress parameters after reperfusion was found in oxLDL for 50.2%, in AOPP for 38.3% and in perOx for 55.2% of all STEMI patients on day 2. There were no significant differences in CMR parameters when comparing patients according to the rise or fall of oxidative stress parameters, except for microvascular obstruction (MO) and AOPP (p=0.043, Table 1). In multivariable stepwise regression analysis for prediction of MO including AOPP, TIMI flow after PCI, diabetes mellitus, pain to balloon time and TIMI risk Score only diabetes remained a significant predictor (Beta 0.22; p=0.004). AOPP values after PCI, on day 3 or 4 after PCI were also no independent predictor of MO.

**Table 1:** Table 1

Day 2	perOx≤1	perOx>1	p	AOPP≤1	AOPP>1	p	oxLDL≤1	oxLDL>1	p
Infarct size %LV	18.7 (9.4;26.5)	14.2 (8.9;23.0)	0.25	16.6 (9.3;25.7)	13.5 (6.0;26.8)	0.29	17.5 (9.3;25.9)	16.2 (7.3;26.5)	0.59
Microvascular obstruction %LV	0.7 (0.2;1.2)	0.6 (0.2;1.6)	0.95	0.8 (0.3;1.4)	0.4 (0.0;1.6)	0.043	0.7 (0.3;1.5)	0.6 (0.0;1.2)	0.28
Area at risk %LV	34.4 (28.8;41.4)	34.6 (28.4;44.1)	0.96	33.9 (27.7;44.5)	35.8 (28.7;43.1)	0.48	35.6 (28.6;43.9)	33.8 (28.3;43.1)	0.45
Myocardial salvage index	45.6 (26.0;73.5)	54.1 (30.2;73.7)	0.32	43.1 (26.4;73.3)	58.4 (29.5;77.1)	0.15	48.5 (28.5;71.4)	44.8 (27.1;75.7)	0.81

## Conclusions

Rise or fall of markers of oxidative stress had no relevant impact on CMR markers of myocardial damage in acute STEMI. Further studies are necessary to elucidate the exact detrimental effect of oxidative stress on myocardial injury in acute reperfused STEMI.

## Funding

N/A.

